# Transcriptome analysis in osmo-primed tomato seeds with enhanced longevity by heat shock treatment

**DOI:** 10.1093/aobpla/plaa041

**Published:** 2020-08-19

**Authors:** Thiago Barbosa Batista, Geysson Javier Fernandez, Tiago Alexandre da Silva, Júlio Maia, Edvaldo Aparecido Amaral da Silva

**Affiliations:** 1 Department of Plant Production, Sao Paulo State University (UNESP), Botucatu, Sao Paulo, Brazil; 2 Institute of Biology, Antioquia University, Medellín, Antioquia, Colombia

**Keywords:** Chaperone molecules, improved longevity, primed seed, seed conservation, seed quality, *Solanum lycopersicum* L, storage

## Abstract

Seed priming is widely used in commercial seeds and its main function is to accelerate and synchronize seed germination. Undesirably, primed seeds show reduced longevity and treatments like heat shock have been shown to improve longevity in primed seeds. Nonetheless, the effect of heat shock treatment on primed seeds at the mRNA level is not known. Thus, the aim of this work was to investigate the effect of heat shock treatment on the longevity of primed tomato (*Solanum lycopersicum*) seeds at the physiological and transcriptome levels. Tomato seeds were primed and dried (control). Alternatively, primed seeds were subjected to heat shock treatment (38 °C/32 % relative humidity) before drying. Germination, vigor and longevity were evaluated. Transcriptome analysis was performed by RNA sequencing (RNA-seq) from biological samples collected immediately after priming and another samples collected from primed seeds followed by the heat shock treatments. The gene expression was validated by quantitative real time PCR (RT-qPCR). We showed that applying heat shock treatment after priming increased germination speed, enhanced seed longevity and preserved the vigor during storage of primed tomato seeds. Through transcriptome analysis, 368 differentially expressed genes were identified, from which 298 genes were up-regulated and 70 were down-regulated. We showed the increase of mRNA levels of *HEAT SHOCK FACTOR*-like and *HEAT SHOCK PROTEIN*-like chaperone genes, suggesting the involvement of the proteins coded by these transcripts in the enhancement of longevity in primed tomato seeds. The heat shock treatment after priming enhances and preserves the vigor of tomato primed seeds during storage. In addition, improves seed longevity through the increase in the expression of transcripts related to protection by response to stress.

## Teaser

Primed seeds show reduced longevity and treatments like heat shock have been shown to improve longevity. In this study, we performed physiological and transcriptome assays in primed tomato seeds with and without heat shock treatment. We showed that primed seeds plus heat shock have enhanced longevity. In addition, we showed the increase of mRNA levels of HSF-like and HSP-like chaperone genes, suggesting the involvement of these proteins coded by these transcripts in the enhancement of longevity in primed tomato seeds. This study expands information regarding the molecular mechanisms that may be involved in seed longevity.

## Introduction

Tomato seeds often exhibit slow and uneven seed germination resulting in propagation problems. To mitigate this problem, researchers and seed companies employ a technique known as priming. The priming technique consists of a series of treatments with controlled amounts of water or osmotic solutions that allow the activation of the germination metabolism without allowing radicle protrusion.

Priming is widely used in vegetable seeds favouring the germination speed and emergence and, thus contributing to the formation of more vigorous seedlings (Asgharipour and Rafiei 2011; Batista *et al.* 2015; Barboza da Silva and Marcos-Filho 2020). In addition, priming also increases the tolerance of seeds and seedlings to biotic and abiotic stresses (Amooaghaie *et al.* 2010; Chen *et al.* 2012; Worrall *et al.* 2012; Chen and Arora 2013; Barboza da Silva and Marcos-Filho 2020). Although priming is widely used in tomato seed lots, the longevity (viability maintenance) of primed seeds is significantly reduced, causing problems in their storage (Argerich *et al.* 1989; Liu *et al.* 1996).

Longevity is defined as the capacity of a seed to remain viable for long periods during dry storage (Sano *et al.* 2016) and its physiological and molecular aspects have been reviewed (Rajjou and Debeaujon 2008; Sano *et al.* 2016). Seed longevity is acquired after the desiccation tolerance and glassy state formation in the seeds (Bewley *et al.* 2013). The glassy state is the transition from liquid cytoplasm to a high viscosity liquid and thus promotes the reduction of cellular mobility and molecular diffusion which prevents chemical reactions that can damage tissues, and thus ensures the longevity extension (Buitink and Leprince 2004; Ballesteros and Walters 2011; Leprince *et al.* 2017). Compounds that accumulated during seed maturation, such as non-reducing sugars (sucrose and raffinose), are involved in the formation and maintenance of a glassy state and these sugars accumulate as the seed water content decreases (Bewley *et al.* 2013; Leprince *et al.* 2017). Late abundant embryogenesis proteins and small heat shock proteins act in conjunction with sugars in the formation of the glassy state (Bewley *et al.* 2013; Kaur *et al.* 2016). In soybean seeds, the increase in transcript levels of *HEAT SHOCK PROTEINS* (*HSP*) and *SMAL HEAT SHOCK PROTEINS* (*sHSP)* genes were correlated with seed longevity (Lima *et al.* 2017).

In addition to the mechanisms derived from the glassy state, the ability to remain viable during storage is also associated, among other factors, with DNA and protein repair systems as indicated by a marked presence of proteins such as DNA ligases (Waterworth *et al.* 2010) and Protein l-isoaspartil methyltransferase (PIMT) (Ogé *et al.* 2008). It was observed that overexpression of *OsHSP18.2*, a class II cytosolic *HSP*, decreases the accumulation of reactive oxygen species (ROS) and allows increase in germination rates after controlled deterioration in *Arabidopsis* seeds (Kaur *et al.* 2015), which indicates the detoxification of ROS during storage.

Thus, primed seeds have mechanisms related to longevity, mentioned previously, impaired by the priming treatment. For instance, it was showed that in primed rice seeds the loss of longevity is associated with the reduced metabolism of starch (Hussain *et al.* 2015; Wang *et al.* 2018), accumulation of malondialdehyde and the decrease in the activities of antioxidant enzymes (Wang *et al.* 2018). In addition, the advancement of the cell cycle from priming acts to reduce longevity, as demonstrated by Sano and Seo (2019) in *Arabidopsis* seeds.

The maintenance of viability during storage is important for preservation of seed quality and the capacity for seedling establishment in the field, which highlights the importance of longevity in primed seeds. Thus, alternatives to extend longevity in primed seeds have been studied. Bruggink *et al.* (1999) demonstrated that water deficit and heat shock after priming in pepper seeds with high water content promotes greater tolerance to the stress imposed by controlled deterioration. Gurusinghe *et al.* (2002) enhanced longevity in primed tomato seeds by the use of heat shock treatment at 37 °C for 2, 3 and 4 h. In the same study, the authors also showed a positive correlation between heat shock treatment, longevity and the accumulation of BiP proteins (78 kD Binding Protein) which is a member of the heat-shock protein family. Although this enhancement of longevity was observed, little is known about the effect of heat shock treatment in primed seeds at the molecular level and what are the mechanisms that govern the enhancement of longevity in primed tomato seeds after heat shock. Therefore, we hypothesize that the application of heat shock treatment on primed tomato seeds induces the expression of genes which code for proteins associated with protection of RNA, DNA and other proteins which are essential for longevity in primed tomato seeds. Thus, to investigate our hypothesis, the aims of the study were: (1) to enhance seed longevity by heat shock treatment in primed tomato seeds, (2) to determine the consequences of the heat shock on primed tomato seeds, and (3) to investigate transcriptomic approach in primed tomato seeds with enhanced longevity promote by heat shock treatment.

## Methods

### Seed production

Seeds of *S. lycopersicum* from the LA1509 access, donated by the Tomato Genetics Resource Center (https://tgrc.ucdavis.edu/), were sown in a tray with commercial organo-mineral substrate. At 28 days, 10 plants were transplanted (45 cm × 1 m) to oxisoil in a polytunnel subjected to local environmental conditions in Botucatu-Brazil in the 2017. The climate conditions were considered no-stress and the average temperature was 21.9 °C. Fertilization during the crop cycle was performed based on soil analysis and drip irrigation was used for plants watering. The fruits were harvested as they matured (red fruits without the presence of green coloration). The fruits were cut with the aid of a knife and the seeds extracted by hand. Following the extraction, the seeds were treated with a sodium hypochlorite solution with 9 % of initial concentration at a ratio of 1:1 with seeds plus the mucilage extracted, for a period of 30 min to remove mucilage adhered to the seeds. The seeds were then washed in running water and placed in forced air convection drying oven at 32 °C/32 % relative humidity (RH) for a period of 24 h after which they reached 0.08 ± 0.01 g H_2_O/g DW^-1^ (grams of water per gram of dry weight) of water content. We measured the water content according to the International Seed Testing Association (2007). The seeds were then stored in glass hermetic pots and kept in at 10 °C and 64 % RH until the start of the experiments.

### Priming treatment

According to established protocol previously, seeds were placed into tubes containing 15 mL of a polyethylene glycol (PEG) 6000 solution with an osmotic potential of -1.0 MP at 20 °C for 60 h in the dark. To avoid the lack of oxygen in solution during incubation, holes were made in the tube caps. Subsequently, the tubes were placed in a mixer (Multifunctional mixer MR-II model-Biomixer) to shake the solution throughout the incubation period. The concentration of the PEG solution was monitored daily. After priming treatment seeds were washed in running water during one minute and excess water was removed with paper towels. After the priming treatment the seed water content was ±1.08 g H_2_O/g DW^-1^. We osmo-primed 100 seed per tube until the quantity of 2.800 osmo-primed seeds was obtained.

### Heat shock treatment

Immediately after priming, part of primed seeds (1.400 seeds) were placed over paper towel and subjected to the heat shock treatment by exposing the seeds to an environment of 38 °C/32 % RH, in an oven with air circulation for 2 h.

### Drying after treatment

Primed seeds, with and without heat shock treatment, after respective treatment, were placed over paper towel and kept for up to 24 h (22 h for the heat shock treatment group) at 20 °C/60 % ±2 % RH, after which the seeds reached the moisture content of 0.09 ± 0.01 g H_2_O/g DW^-1^.

Following drying, the seeds were stored in glass hermetic pots, and placed under 10 °C and 64 % RH and at 10 days after treatment, the physiological assays were performed.

### Physiological assays

#### Seed germination and vigor

 Four replications of 50 seeds were germinated in 9 cm Petri dishes with substrate of paper towel moistened with distilled water equivalent to 2.5 times its weight, at 25 °C, under 8 h of light and 16 h in the dark. The length of the primary root, ≥2mm was used as the germination criterion. Data collection was done in different times after sowing; and ended when the germination rate reached 100 % or at 14 days. Seed vigor was determined by the calculation of the time to 50 % of germination (t50) through the analysis of cumulative germination data using the curve fitting module of the Germinator software package (Joosen *et al.* 2010).

#### Longevity

We used ageing protocol to assess seed longevity, in which the seeds were placed in a support over a saturated solution of NaCl (75 % RH) at 35 °C in glass bottles hermetically sealed. During storage, the water content of *S. lycopersicum* seeds stabilized at 0.10 ± 0.007 g H_2_O/g DW^-1^, corresponding to ±9.5 % on wet basis. At different time spans, seeds were imbibed and viability was assessed using the germination assay as described earlier. The different time spans were carried out considering the viability loss behaviour of each treatment group during storage. The viability data were transformed into probit to determine the moment when the germination was reduced in half (p50), by using the equation: *v = (Ki - p)/σ*, according to Ellis and Roberts (1980). Where: *v =* viability in days, *Ki =* initial germination in probit values, *p =* expected death over time and *σ =* slope of the curve. We determined the seed vigor previously the p50 at 45 days, by calculating the t50 as described earlier.

#### Statistical analysis from physiological assays

We performed the normality and homogeneity of the data through the Shapiro–Wilk test and Bartlett test, respectively. The data fulfilled the assumption for normality and homogeneity. Thus, the vigor (t50) and longevity (p50) data of primed seeds with and without heat shock treatment were compared by *t*-test at 0.05 confidence level. The sigmoidal behaviour was adjusted using the Boltzmann equation parameters.

### RNAseq and RT-qPCR analysis

#### Preparation and processing of mRNA-Seq libraries

Three biological samples of 100 seeds each were collected immediately after priming and another three samples of 100 seeds each were collected from primed seeds followed by the heat shock treatments. They were all stored at -80 °C. Total RNA was extracted using the NucleoSpin^®^ RNA plant Kit (Macherey-Nagel, Düren, GER) and strictly followed the manufacturer’s instructions. The quality and quantification of the total RNA samples were evaluated in 2100 Bioanalyzer and biological samples with RIN ≥7.2 were used for the later stages. The RNA-Seq was performed in a HiSeq 2000 Sequencing System Platform (Illumina, USA) using the services of the Central Laboratory of High Performance Technologies (LaCTAD-Campinas-Brazil). The protocol used to construct the library and sequencing is available at: http://goo.gl/hyslD. Sequencing protocol included the preparation of total RNA, followed by fragmentation and purification of the messenger RNA. The next step was the amplification for the construction of cDNA libraries: hybridization and binding of adapters, reverse transcription, cDNA purification and, finally, amplification and quantification of the amplified cDNA. This cDNA was diluted and used to generate clusters (amplification of specific fragments), and subsequently sequenced. Constructed libraries were 100 base pair (bp) paired-end sequenced. The data output in fastq file format contained sequence information, including the sequencing quality (Phred quality score). Average Phred scores of ≥20 per position were used for the alignment.

#### Read alignment and differentially expressed genes

Paired-end reads for mRNA were mapped to the *Solanum lycopersicum* release 39 reference genome using the default parameters of TopHat2 (Kim *et al.* 2013). Counts for RefSeq genes were obtained using HTSeq (Anders *et al.* 2015) and DESeq2 (Love *et al.* 2014) was used to normalize expression counts. The changes in gene expression were considered statistically significant when fold change ≥2 and *P*-values ≤ 0.05. The RNAseq data was deposited in NCBI (BioProject PRJNA562700: https://trace.ncbi.nlm.nih.gov/Traces/sra/?study=SRP220280).

The analysis of principal components was made using all the genes expressed on the RNA seq data. The normalized count per gene was used and transformed to Z-score. This matrix was used was used to perform the PCA. For plotting the PCA results, we used the principal component one and two. The heatmap was generated using the normalized counts of the differentially expressed genes. Then we transformed it to z-score and plotted it using the package pheatmaps of R.

#### Gene ontology enrichment analysis

Was performed using the PANTHER classification system (Mi *et al.* 2017), using a hypergeometric test with a Benjamini and Hochberg False Discovery Rate correction. A *P*-value cut-off of 0.05 and Fold enrichment of 2 was used to identify enriched processes.

#### cDNA synthesis and RT-qPCR

 cDNA was synthesized from 10 µL of total RNA extracted and stored at -80 °C, using a High Capacity cDNA Reverse Transcription kit (Applied Biosystems, Victoria, AUS) following the manufacturer’s instructions. For a reaction of 20 µL, 5.8 µL of RT Supermix and 10 µL RNA were used and the volume was completed with Nuclease-free water. The reactions were incubated in a thermocycler at 25 °C for 5 min for the initial activation of the enzyme and at 37 °C for 2 h for cDNA synthesis, followed by inactivation of the enzyme at 85 °C for 5 min and the cycle was concluded at a constant temperature of 4 °C.

For this study, genes target were selected based on differential expression of RNA-seq data. Genes with fold change between control and treated equal to 1 and with coefficient of variance between samples <20 %, were selected as reference genes for normalizing the RT-qPCR data. Forward and reverse primers used for these genes are listed in Supporting Information—Table S1.

For the design of the primers target and normalizers, we used the *PerlPrimer* Software (http://perlprimer.sourceforge.net) for amplification between 80 and 150 bp. Primer efficiency was calculated, after the RT-qPCR, through the LinRegPCR program (Ruijter *et al.* 2013). The efficiency of the primers was close to 1.8 and showed an *R*^2^ of approximately 1.0.

We performed the gene expression on a thermocycler Eco Real-Time (Illumina) with SYBR Green qPCR ReadyMix (Sigma Aldrich). For a reaction of 10 µL, 5 µL of SYBR Green, 1 µL of cDNA and 0.25 µL of each primer were used and the volume was completed with Nuclease-free water. The amplification was performed with initial step of incubation at 50 °C for 2 min, followed by denaturation at 95 °C for 2 min, 45 cycles with denaturation at 95 °C for 10 s and annealing at 60 °C for 1 min. At the end of the process, the melting curve was performed following these steps: 15 s at 95 °C, 65 °C and 95 °C, respectively. Data were analysed with EcoStudy program version 5.0 (Illumina).

Relative expression levels were calculated by comparative 2^-ΔΔCt^ method using a geometric mean of two reference genes, *60S RIBOSOMAL PROTEIN* and *TUBULIN ALPHA CHAIN* (in the RT-qPCR these genes showed no variation greater than 0.8 in Cq value), comparing the treated sample with the control sample. The REST^®^ program was used to perform validation of the relative expression using the Pair-Wise Fixed Reallocation Randomization Test (Pfaffl *et al.* 2002).

## Results

### The influence of the priming treatment and heat shock on the physiological quality of *S. lycopersicum* seeds

**Figure 1.**
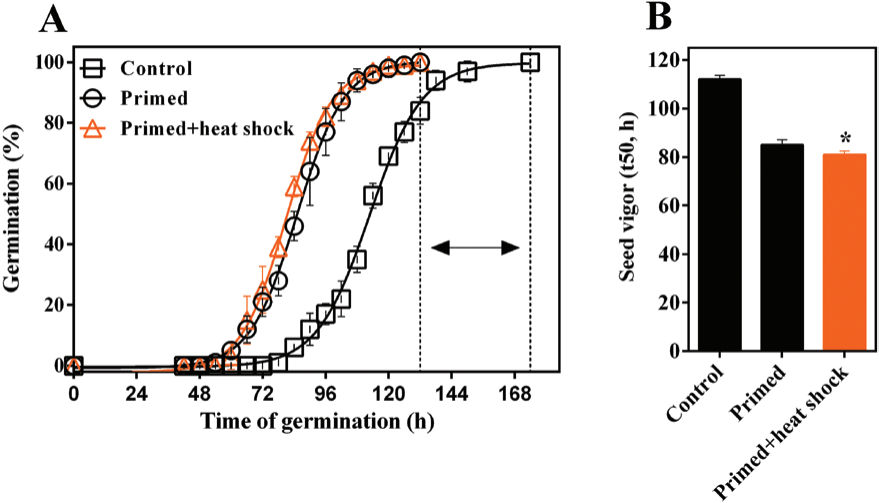
Priming and heat shock treatment improve physiological performance in *S. lycopersicum* seeds. Germination curves (A) and seed vigor—t50 (B) in *S. lycopersicum* seeds control non-primed, primed and primed+heat shock treatment (38 °C/32 % RH).. *Significance difference (*P* ≤ 0.05) by *t*-test between primed and primed+heat shock samples (*n* = 4). Error bars show standard deviation.

The priming treatment at -1.0 MP of PEG solution accelerated germination of *S. lycopersicum* seeds (Fig. 1A), thus allowing the conditioned seeds to reach maximum germination quicker, while the seeds of the control treatment took 42 h more to show the same percentage of germination (indicated by arrows in Fig. 1A). The priming treatment increased the speed of germination, because it reduced the t50 in 27 h in relation to non-primed seeds (Fig. 1B). In addition, there was significant (*P* = 0.032) benefit from the heat shock treatment (38 °C/32 % UR) after priming, since it reduced the t50 in 4 h in relation to the treatment with PEG -1.0 MP (Fig. 1B).

Primed seeds had a water content of 1.08 g H_2_O/g DW^-1^ immediately after priming and after the heat shock treatment it was reduced to 0.08 g H_2_O/g DW^-1^, i.e. a loss of ±1 g H_2_O/g DW^-1^ in a period of 2 h at 38 °C. Thus, the heat shock treatment was characterized as fast drying and it did not reverse the priming effect observed (Fig. 1).

**Figure 2.**
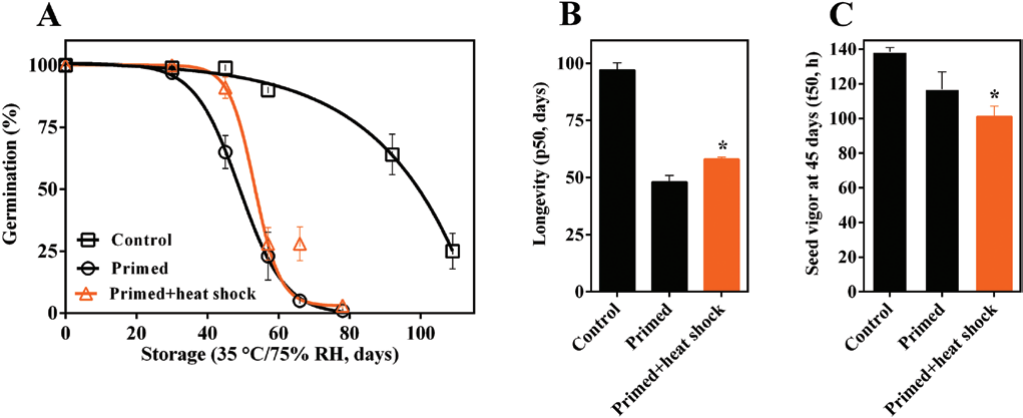
Priming decrease longevity in *S. lycopersicum* seeds while it is enhanced by heat shock treatment. Germination during storage (A) longevity (B) and seed vigor—t50—at 45 days in *S. lycopersicum* seeds control non-primed, primed and primed+heat shock treatment (38 °C/32 % RH). Germination during storage was fitted with Boltzmann sigmoid [y= top+(bottom-top)/1+[v50−(x/slope)]].*Significance difference (*P* ≤ 0.05) by *t*-test between primed and primed+heat shock samples (*n* = 4). Error bars show standard deviation.

Although primed seeds showed superior speed of germination (Fig. 1B), they showed faster loss of viability during storage at 35 °C/75 % RH, in relation to seeds that were not subjected to the priming treatment (Fig. 2). Applying heat shock treatment at 38 °C/32 % RH after priming promoted a moderate and significant retention of the germination capacity during storage in comparison to the seeds that were only primed (Fig. 2B).

The p50 (time to lose of 50 % germination during storage) of the control non-primed seeds was of 97 days and after priming it was 48 days. However, the p50 increased in 10 days moving from 48 to 58 days after the heat shock treatment in the primed seeds (Fig. 2B). This shows a significant (*P* < 0.001) enhancement of seed longevity (Fig. 2B). Moreover, the heat shock treatment preserved a significantly (*P* = 0.028) low germination time (t50) during storage, since it reduced the t50 in 15 h in relation to the treatment with PEG -1.0 MP at 45 days of storage. Thus, the heat shock treatment not only enhanced longevity in primed seeds but also preserved the seed vigor during storage (Fig. 2C).

### Identification of transcripts in primed seeds subjected to heat shock treatment

To identify transcripts related to the enhancement of longevity in primed seeds subjected to heat shock treatment, we performed RNAseq analysis in primed seeds and in primed seeds plus heat shock treatment. The alignment of the mapped readings was ≥82 % in the reference genome of *S. lycopersicum* [see Supporting Information—Table S2] and allowed us to verify the expression of 7560 genes.

**Figure 3.**
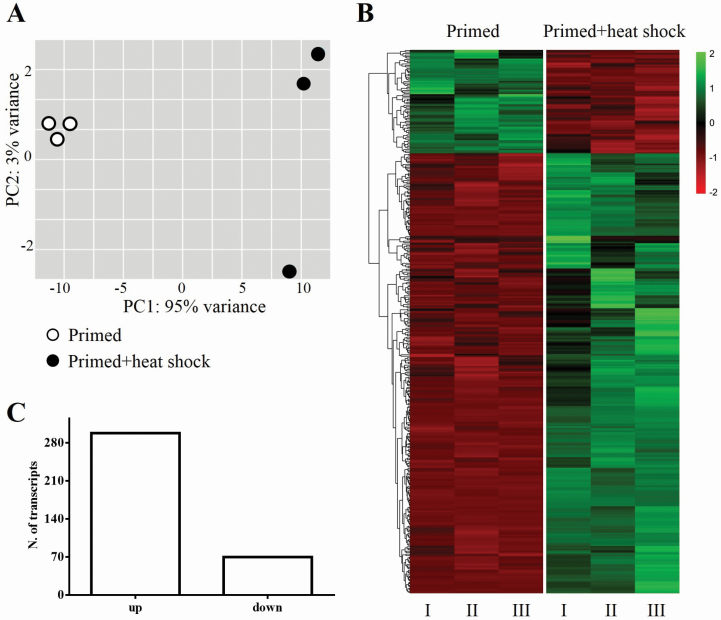
Seed expression profile of *S. lycopersicum* primed and primed+heat shock treatment (38 °C/32 % RH). (A) Principal component analysis of expressed genes. (B) *Heatmap* of differentially expressed genes. (C) Number of up- and down-regulated transcripts of differentially expressed genes.

The principal components analysis of the mapped genes demonstrated a difference between the groups of primed seeds and primed seeds subjected to heat shock, as well as a higher heterogeneity in the regulation of the transcriptome in the samples submitted to the heat shock treatment (Fig. 3A) which occurred due to the presence of an outlier. The analysis of these 7560 genes revealed 368 differentially expressed genes (false discovery rate ≤0.05 and fold change ≥2) in *S. lycopersicum* seeds, of which 298 genes were up-regulated and 70 were down-regulated (Fig. 3B and C, see Supporting Information—Table S3).

The enrichment analysis of the gene ontology biological process revealed that the most enriched biological processes are related to the ‘stress’ imposed by the heat shock treatment at 38 °C/32 % RH, as the response to heat. Interestingly, gene ontology biological process linked to protein folding was highly enriched and the genes related to this process represented almost 2 % of the total number of differentially expressed genes (Table 1).

**Table 1.** Enrichment of gene ontology of the complete biological process of differentially expressed genes (DEG) found in seeds of *S. lycopersicum* submitted to heat shock treatment at 32 % RH/38 °C.

Gene ontology of the biological process complete	Number of genes	DEG	Expected	Fold Enrichment	FDR
Response to heat	19	5	0.18	27.66	3.26E-03
Response to temperature stimulus	45	8	0.43	18.69	1.29E-04
Protein folding	168	11	1.6	6.88	2.21E-03
Response to abiotic stimulus	265	12	2.52	4.76	1.32E-02

FDR = false rate discovered.

Among the genes identified, *DNAJ PROTEIN HOMOLOG* is found in all enriched pathways (Table 2; see Supporting Information—Table S4). Our analysis revealed the presence of *HSP*, such as *17.4 kDa CLASS III HEAT SHOCK PROTEIN* and *HEAT SHOCK PROTEIN 90* with Log2 expression fold change ≥5, verified in the heat response pathway and temperature stimulus, protein folding pathway, respectively (Table 2; see Supporting Information—Table S4). Interestingly, our analysis revealed the gene *SMALL HEAT SHOCK PROTEIN PRECURSOR* (*er-sHSP*) and *15.7 kDa HEAT SHOCK PROTEIN* with Log2 Fold change ≥1.30 and 1.84, respectively (Table 2; see Supporting Information—Table S4). These genes have not been reported in other studies of seed longevity.

**Table 2.** Description and Log2 Fold change of genes present within enriched pathways of complete biological process.

Gene Identification	Description	Log2 Fold change
Solyc03g123540	*17.4 kDa CLASS III HEAT SHOCK PROTEIN*	5.53
Solyc06g036290	*HEAT SHOCK PROTEIN 90*	5.04
Solyc10g084170	*BAG FAMILY MOLECULAR CHAPERONE REGULATOR 5*	4.2
Solyc03g007890	*HEAT SHOCK PROTEIN 83*	4.14
Solyc06g072430	*UNCHARACTERIZED PROTEIN*	3.79
Solyc02g077670	*DNAJ PROTEIN HOMOLOG 1*	3.37
Solyc11g071830	*DNAJ PROTEIN HOMOLOG*	3.06
Solyc02g088610	*HEAT SHOCK PROTEIN (HSP100/CLPB)*	2.47
Solyc11g020040	*HEAT SHOCK PROTEIN 70*	2.08
Solyc09g091030	*BETA-AMYLASE*	1.97
Solyc03g007650	*DNAJ LIKE PROTEIN*	1.90
Solyc04g014480	*15.7 kDa HEAT SHOCK PROTEIN*	1.84
Solyc09g092690	*PEPTIDYL-PROLYL CIS-TRANS IS* *OMERASE FKBP65*	1.66
Solyc02g036370	*PROTEIN REVEILLE 1*	1.60
Solyc02g084850	*ABSCISIC ACID AND ENVIRONMENTAL STRESS-INDUCIBLE PROTEIN TAS14*	1.59
Solyc11g020330	*SMALL HEAT SHOCK PROTEIN PRECURSOR*	1.30
Solyc12g005420	*B-BOX ZINC FINGER PROTEIN 22-LIKE*	1.21
Solyc12g015880	*MOLECULAR CHAPERONE HSP90-1*	1.08
Solyc12g014470	*GRPE PROTEIN HOMOLOG 2*	1.04
Solyc03g119690	*PROTEIN ASPARTIC PROTEASE IN GUARD CELL 1*	-1.23

**Figure 4.**
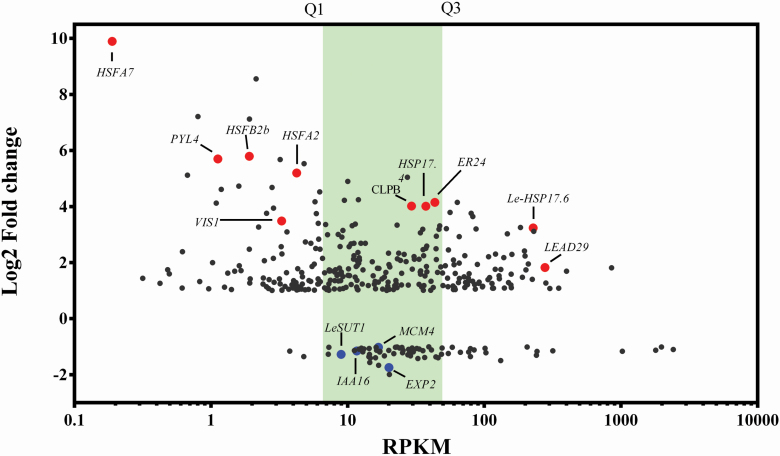
Dispersion between abundance (RPKMs, x-axis) and degree of expression (log2 fold change, y-axis) of up- (●) and down-regulated genes (●) identified. Each point represents differentially expressed genes. Green shade indicates division between low, medium and high abundance, defined using quartiles (Q1 and Q3).

We used a dispersion graph that integrates the degree of regulation (Log2 Fold change) and abundance in reads per kilobase million (RPKM) to identify changes between the regulation and abundance of the transcripts that were outside the enriched pathways (Fig. 4).

Thus, among the up-regulated genes, the transcripts of the *HEAT SHOCK FACTOR* (*HSF*) family such as *HSFA7* (*HEAT SHOCK FACTORA7,* Solyc09g065660.2), *HSFB2b* (*HEAT SHOCK FACTORB2B,* Solyc08g080540.2) and *HSFA2* (*HEAT SHOCK FACTORA2,* Solyc08g062960.2) were identified in the area of low abundance; however, with Log2 fold change ≥5 (Fig. 4). Transcripts from the family of *HSP* such as *VIS1* (Solyc05g014280.2), *HSP17.4* (Solyc08g062340.2) and *Le-HSP17.6* (Solyc08g062450.1) showed variable abundance and Log2 fold change ≥3 (Fig. 4). We identified the transcript *ER24* (*ETHYLENE-RESPONSIVE TRANSCRIPTIONAL COACTIVATOR*, Solyc01g104740.2) with Log2 fold change ≥4 and *LEAD29* (*LATE EMBRYOGENESIS ABUNDANT PROTEIN D-29*, Solyc12g098900.1) with Log2 fold change ≥1.82, in the area from intermediate to high abundance, respectively (Fig. 4).

Among the down-regulated genes, we identified the transcript of *EXP2* (*EXPANSIN*, Solyc06g049050.2), *IAA16* (*AUXIN RESPONSIVE PROTEIN*, Solyc01g097290.2) and *MCM4* (*DNA REPLICATION LICENSING FACTOR*, Solyc01g110130.2) in the areas of intermediate abundance (Fig. 4).

**Figure 5.**
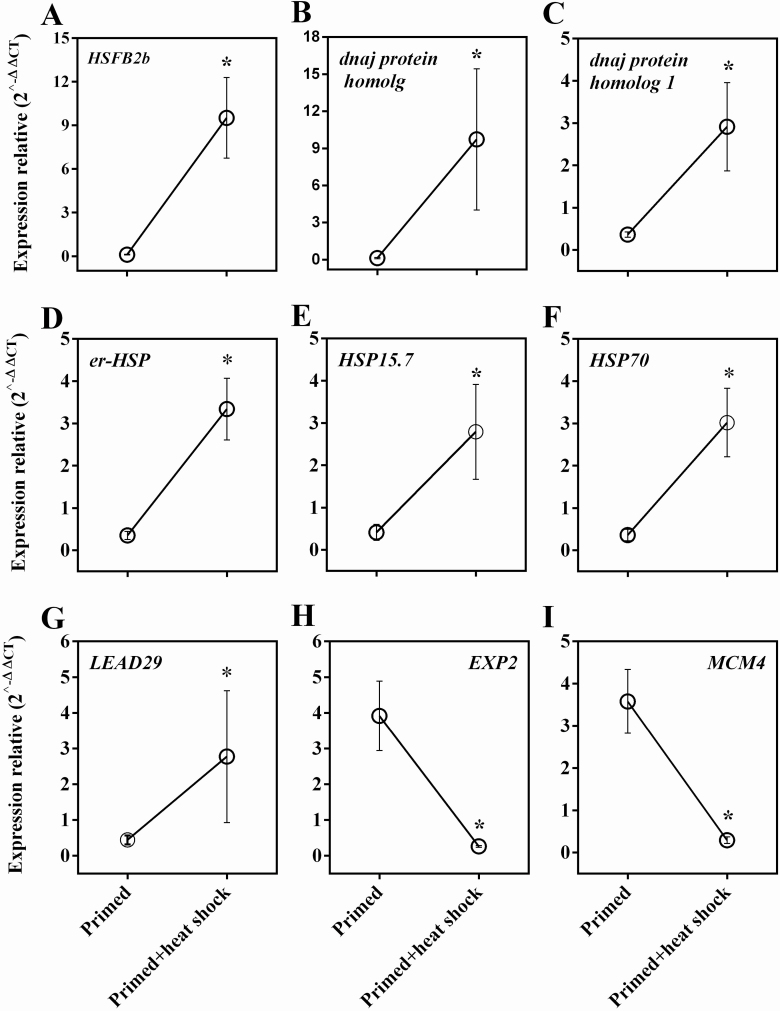
RT-qPCR of selected genes from primed and primed+heat shock treatment (38 °C/32 % RH) validated the RNAseq data. *Significant difference of expression between primed+heat shock and primed samples using the Pair-Wise Fixed Reallocation Randomization Test (*n* = 3). Error bars show standard deviation.

The RT-qPCR analysis allowed the validation of genes associated with seed longevity (Fig. 5). Through this, it was possible to verify the increase in the expression of genes related to protection in samples subjected to heat shock treatment after priming, such as *HSFB2b* and chaperone molecules such as *sHSP* and *HSP* that were up-regulated in the transcriptome (Fig. 5A–G). In addition, a decreased expression of two down-regulated genes related to cell division and expansion was verified (Fig. 5H and I).

## Discussion

Seed priming is an important technology that improves speed germination and uniformity of seed lots. However, primed seeds have a short lifespan. Here, we demonstrated that a heat shock treatment of 38 °C/32 % RH after priming is able to enhance longevity in primed tomato seeds. In addition, we also showed that the enhancement of longevity is associated to a change in the transcriptome profile that leads to an increased expression of genes related to protection by response to stress.

First, we showed that the priming protocol established (PEG 6000 with an osmotic potential -1.0 MP at 20 °C during 60 h) improved seed quality of *S. lycopersicum*, by increasing the speed of germination (Fig. 1). However, although beneficial to speed germination and consequently the vigor of *S. lycopersicum* seeds, the priming treatment accentuated the loss of longevity during storage compared with non-primed seeds (Fig. 2A). The short longevity of primed seeds was also verified in lettuce seeds (Hill *et al.* 2007), *Impatiens* and pepper (Buitink *et al.* 2000). Thus, we decided to investigate whether the heat shock treatment after priming would enhance longevity in primed tomato seeds as indicated by Gurusinghe *et al.* (2002). Indeed, the heat shock treatment performed at 38 °C/32 % RH for 2 h did not affect the final germination (100 %) in primed tomato seeds (Fig. 1A) and when carried out before storage at 35 °C and 75 % RH allowed a significant enhancement of longevity compared with the conventional priming treatment (Fig. 2A and B). In practical terms, we need to highlight that in ideal storage conditions, the longevity of primed tomato seeds treated by heat shock can increase by perhaps months or years; which can be consistent considering that tomato seeds have longevity (p50) of around 10 years as showed by Fleming *et al.* (2019).

Recently, Fleming *et al*. (2019) showed that during storage the loss of germination is sigmoidal and asymptomatic initially, whereas the loss of integrity of RNA is linear and precedes the loss of longevity. In our study, the loss of germination during storage was sigmoidal (Fig. 2A). The integrity of the RNA, however, was not measured during storage, but initially the heat shock did not affect the integrity of the RNA (RIN > 7.2) used for analysis of the transcriptome. Therefore, it is possible to infer that our methodology can be used in the conservation of primed seeds to extend the longevity and preserve the integrity of the transcripts, which can be important for maintaining the longevity of seeds as indicated by physiological assays.

Following the priming analyses, we performed a transcriptome study to identify the transcripts associated with the retention of longevity promoted by the heat shock treatment. In our RNAseq data, the heat shock treatment induced the expression of transcripts associated with response to stress (Tables 1 and 2).

We found the expression of *LEA* protein transcripts and a remarkable enrichment of *sHSP* transcripts after the heat shock treatment, which is possibly related to their chaperone function to prevent aggregation and denaturation of proteins (Lee *et al.* 1997) as reported for *sHSP18.2* by Kaur *et al.* (2015) in rice and *Arabidopsis* seeds. *HEAT SHOCK PROTEINS* are molecular chaperones expressed in response to stresses and protect proteins against stress conditions (Lee *et al.* 1997; Ma *et al.* 2019). Certainly, the presence of these transcripts is due to the enrichment of stress-related pathways (Table 1) through the heat shock treatment.

Gurusinghe *et al.* (2002) demonstrated an increase in the intensity of BiP proteins in the heat-shock protein family, and the increased expression of these proteins was associated with prolonged longevity of primed tomato seeds subjected to a heat shock at 37 °C for 2, 3 or 4 h after priming. The BiP proteins of the *HSP* class are related to the restoration of proteins damaged by heat and/or incorrect folding (Lee and Vierling 2000), and function as a chaperone in the preservation of the protein structure during dehydration or protein reactivation damaged during the acquisition of water and drying (Gurusinghe *et al.* 2002). The *DNAJ PROTEIN HOMOLOG* gene observed in the present study (Table 2; Fig. 5) also has a chaperone function reported, induced by heat shock, which plays a fundamental role in plant growth (Fan *et al.* 2017) and induction of stress resistance (Bekh-Ochir *et al.* 2013). In our study, we also found *sHSP* that are associated to germination performance mentioned by other authors such as *sHSP17.6* (Bettey and Finch-Savage 1998; Lima *et al.* 2017). However, our RNAseq data reinforce the action of the protection molecules through heat shock treatment for seed longevity as mentioned before.

We observed a reduction in the expression of *EXP2* in seeds with enhanced seed longevity thought transcriptomic approach and RT-qPCR analysis. This result is consistent with that found by Sano *et al.* (2017) in primed *Arabidopsis* seeds, in which cell wall modification-related genes including expansin were less expressed in longer-longevity seed populations following priming.

The HSF transcripts are important transcription factors that regulate the expression of several genes responsive to stress and play a key role in abiotic stress tolerance (Guo *et al*. 2016). This justifies the presence of *HSFA3*, *HSFA2* and *HSFB2b*, as revealed by our transcriptome studies in seeds subjected to heat stress after priming (Fig. 4). Furthermore, it extends the possibilities of relating these transcripts to longevity, since only *HSFA9* has been reported as seed-specific (Kotak *et al.* 2007). Here we showed that *HSFB2b* is expressed in dry tomato seeds after the heat shock treatment, strongly suggesting a role on seed longevity (Fig. 5A). Through our experiments, it can be inferred that the priming treatment alone is not able to induce the transcripts related to the protection transcripts mentioned here, requiring an additional heat shock treatment for increased longevity of priming seeds. Therefore, the methodology used here is another excellent system for understanding the molecular mechanisms associated with seed longevity.

In addition, heat shock treatment after priming preserved the seed vigor of *S. lycopersicum* during storage (Fig. 2C). Seed vigor decreases after long storage even in ideal conditions, as it was shown for primed bell pepper and rice seeds (Hussain *et al.* 2015; Barboza da Silva and Marcos-Filho 2020), suggesting the importance of the heat shock treatment to preserve vigor after priming.

Heat shock proteins participate in the germination process (Silveira *et al.* 2019) and have been also associated with seed vigor (Kaur *et al.* 2015), as reported by Bettey and Finch-Savage (1998) for *sHSP17.6*, which affects seed stress tolerance and, therefore, contributes to seed vigor. Recently, Ma *et al.* (2019) demonstrated that the overexpression of the *AtHSP23.6* and *SlHSP23.8B* gene increases the germination speed in *Arabipdosis* and tomato seeds, respectively. In our study, the germination speed of tomato seeds increased after the heat shock treatment with remarkable accumulation and expression of *HSPs* (Figs 1B and 4; Table 2). The presence of small *HSP* transcripts and the subsequent validation of *HSP*15.7 and *er-sHSP* through the RT-qPCR analysis (Fig. 5) indicate that these molecules can act on seed vigor after priming to preserve vigor during storage (Fig. 1B and 2C), thus preventing the effects associated with deterioration.

 Thus, the results of the present study confirmed that the use of heat shock treatment enhanced longevity in primed seeds. In addition, it improved and preserved seed vigor during storage. This study also expands information regarding the molecular mechanisms that may be involved in seed longevity; and apparently, molecules associated with response to stress are strongly related with it.

## Conclusions

1. The heat shock treatment in primed seeds enhances the longevity of *S. lycopersicum* seeds and contributes to improve germination speed and preserve seed vigor during storage.
2. The increase in the mRNA level of chaperone molecules such as *sHSP*, *HSP* and the transcription factor *HSFB2b* is related to the enhancement of the longevity in primed tomato seeds subjected to heat shock treatment.

## Supporting Information

The following supporting information is available in the online version of this article—

Table S1. Primers sequences used as target and reference genes used in RT-qPCR reactions.

Table S2. Mapping of pared-end reads to the tomato genome.

Table S3. List of genes differentially expressed in response to heat shock compared to the control.

Table S4. List of genes differentially expressed identified in gene ontology enrichment analysis.

plaa041_suppl_Supplementary_Table_S1

plaa041_suppl_Supplementary_Table_S2

plaa041_suppl_Supplementary_Table_S3

plaa041_suppl_Supplementary_Table_S4

## Notes

**Form & Function. Chief Editor**: Kate McCulloh
